# Supplementary material for: Observation of plastic ice VII by quasi-elastic neutron scattering
Source: Nature. 2025 Feb 12;640(8059):662–7. doi: 10.1038/s41586-025-08750-4 (PMC12003197; doi:10.1038/s41586-025-08750-4)
Supplement: Supplementary file 1 — This Supplementary Information file includes Supplementary Figs. 1–9 and contains the following sections: 1 Multiple scattering correction and 2 Simulations and extra Refs. [file 41586_2025_8750_MOESM1_ESM.pdf]

nature portfolio

https://doi.org/10.1038/s41586-025-08750-4

Supplementary information

Observation of plastic ice VII by quasi-elastic neutron scattering

In the format provided by the
authors and unedited

Supplementary Information for: Observation of Plastic Ice VII by Quasi-Elastic Neutron Scattering

Maria Rescigno[1,2], Alberto Toffano[3,4], Umbertoluca Ranieri[1,5], Leon Andriambariarijaona[1], Richard Gaal[2], Stefan Klotz[6], Michael Marek Koza[7], Jacques Ollivier[7], Fausto Martelli[4,8], John Russo[1], Francesco Sciortino[1], Jose Teixeira[9], Livia Eleonora Bove[1,2,6]

[1]Dipartimento di Fisica, Sapienza Università di Roma, Piazzale Aldo Moro 5, Roma, 00185, Italy.
[2]Laboratory of Quantum Magnetism, Institute of Physics, École Polytechnique Fédérale de Lausanne, Lausanne, CH-1015, Switzerland.
[3]School of Mathematics, University of Bristol, Woodland Road, Bristol, BS8 1UG, United Kingdom.
[4]IBM Research Europe, Keckwick Lane, Daresbury, WA4 4AD, United Kingdom.
[5]Centre for Science at Extreme Conditions and School of Physics and Astronomy, University of Edinburgh, Edinburgh, EH9 3FD, United Kingdom.
[6]Institut de Minéralogie, de Physique des Matériaux et de Cosmochimie (IMPMC), CNRS UMR7590, Sorbonne Université, Paris, 75252, France.
[7]Institut Laue Langevin (ILL), 38042 Grenoble Cedex 9, France.
[8]Department of Chemical Engineering, The University of Manchester, Oxford Road, Manchester, M13 9PL, United Kingdom.
[9]Laboratoire Leon Brillouin, CNRS-CEA, Saclay, France.

Contributing authors: maria.rescigno@uniroma1.it; alberto.toffano@bristol.ac.uk ; umbertoluca.ranieri@ed.ac.uk ; leonmarcel.andriambariarijaona@uniroma1.it; richard.gaal@epfl.ch; stefan.klotz@upmc.fr; Koza@ill.fr; ollivier@ill.fr; Fausto.Martelli@ibm.com; john.russo@uniroma1.it;

francesco.sciortino@uniroma1.it ; jose.teixeira@cea.fr;
livia.bove@upmc.fr;

1 Multiple Scattering correction

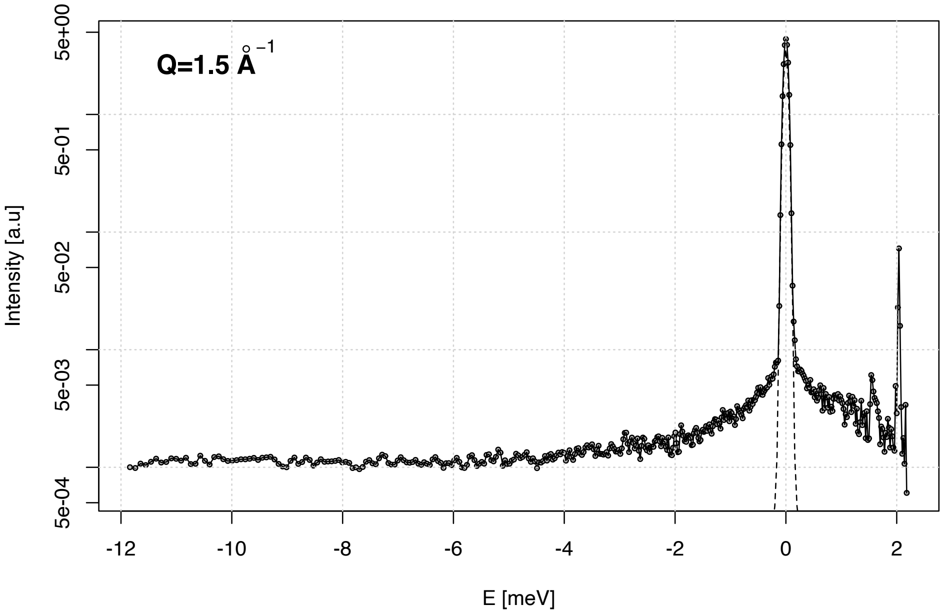

Fig. 1: Full spectrum at 518 K and 5 GPa for Q=1.5 Å^{-1} after data reduction and background subtraction. Secondary peaks are visible between 1 and 2 meV. For this reason, we fitted data up to 1 meV only.

The multiple scattering contribution is recursively estimated using as guess function the $S_0(Q,\omega)$ given by the analysis with one single Lorentzian for three Q values (0.3 Å^{-1}, 1.1 Å^{-1}, 2 Å^{-1}). The sample is described as a cylinder with dimensions given by the gasket dimensions. The gasket contribution is totally elastic in the probed energy range, and its shape is modeled as a hollow cylinder with dimensions given by the external extruded gasket, see figure 2. The simulated single and multiple contributions are evaluated. Multiple contributions are then subtracted to the $S_0(Q,\omega)$ guess, and the new obtained scattering function $S_1(Q,\omega)$ is then used as input for the new simulation. This iterative procedure is repeated till convergence, which is fixed to the equality of $S_i(Q,\omega)$=$S_{i+1}(Q,\omega)$. In figure 3 final results of the procedure showing multiple and single scattering contributions are reported. We observe that at the lower Q values (0.3

Å^{-1}), the multiple signal has the same intensity and HWHM as the single scattering contribution, thus in the measured QENS spectra we expect the multiple scattering contribution to be roughly 50% of the total measured intensity. At 1.1 Å^{-1} the multiple intensity is reduced to 30% of the total signal, while at 2.1 Å^{-1} it is 8%. An example of this subtraction for three selected Q values is shown in figure 4 for a "plastic" point.

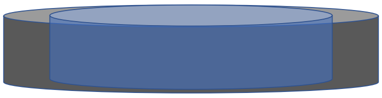

Fig. 2: Geometry for multiple scattering evaluation, with sample in blue and gasket in black.

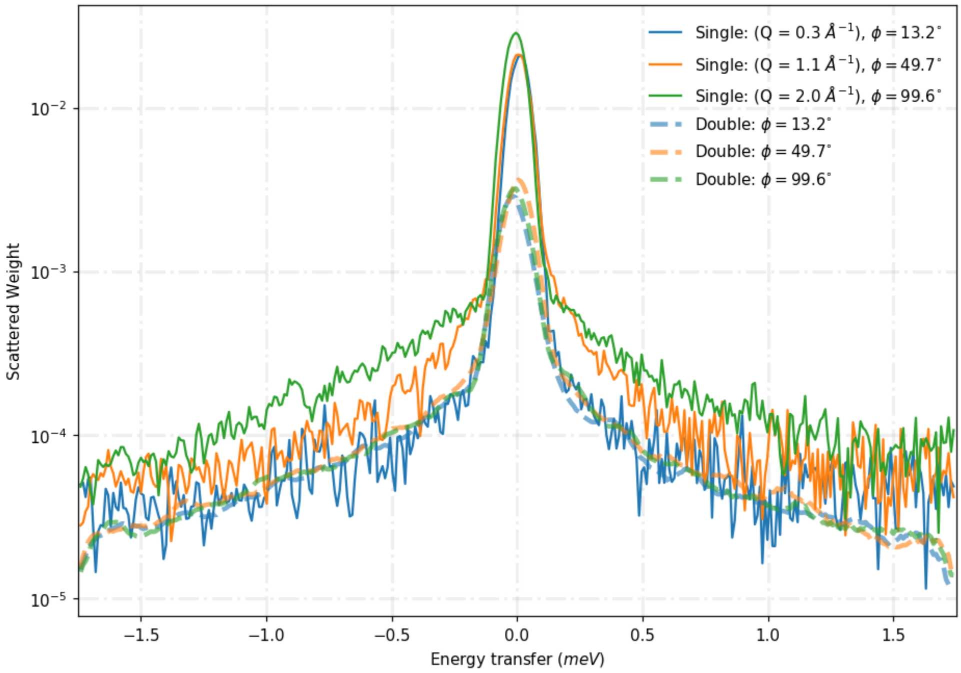

Fig. 3: Results for multiple scattering simulations at Q=0.3, 1.1 and 2 Å^{-1}. Simulations are performed at 5 GPa.

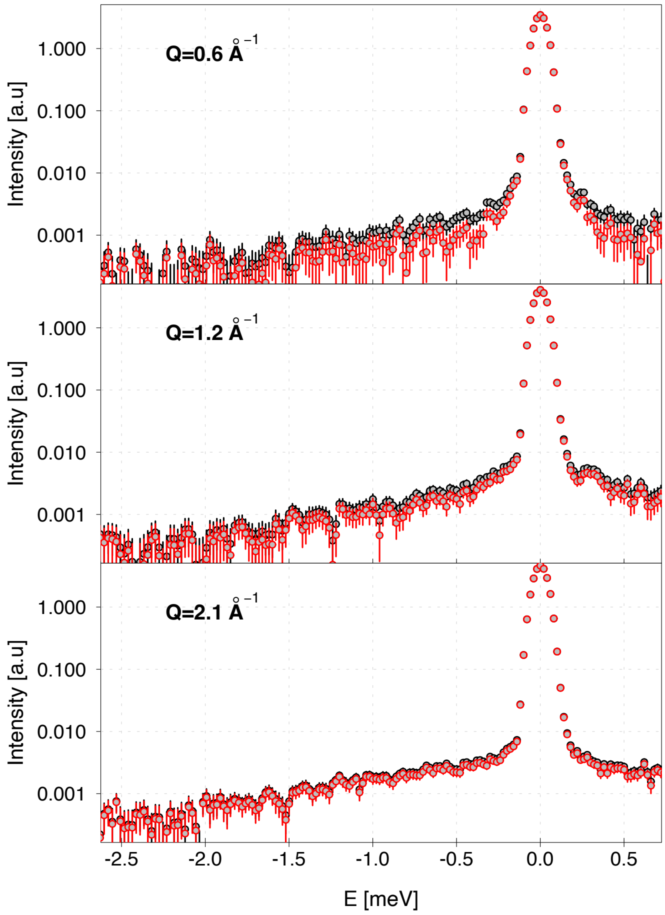

Fig. 4: Spectra at 528 K and 5 GPa for Q=0.6, 1.2 and 2.1 Å^{-1} before (black) and after (red) multiple scattering correction.

2 Simulations

2.1 Decoupling Approximation

For a system of N particles (in our case the hydrogen atoms), the intermediate scattering function (ISF) $F_s(Q,t)$ is computed as the following correlation function:

$$F_s(Q,t) = \frac{1}{N}\left\langle \sum_{j=1}^{N} \exp\left[\imath \mathbf{Q}\cdot(\mathbf{r}_j(t)-\mathbf{r}_j(0))\right]\right\rangle \tag{1}$$

where $\mathbf{r}_j(t)$ is the position of the j-th hydrogen at time t. The ISF is directly connected to the self-dynamic structure factor $S_s(Q,w)$ through the relationship:

$$S_s(Q,\omega) = \frac{1}{2\pi}\int_{-\infty}^{+\infty} F_s(Q,t)e^{-i\omega t}dt \tag{2}$$

Molecular dynamics allows the separation of vibrational and rotational contributions to verify the assumption that these motions are independent. In this framework, and in the absence of translational motion, the ISF (eq. 1) can be re-written as:

$$F_s(Q,t) = F_s^{ROT}(Q,t)F_s^{COM}(Q,t) \tag{3}$$

where F_s^{ROT} and F_s^{COM} represent the rotational (both proper rotations and librations) and vibrational contribution of the centre of mass (COM), respectively. The latter is determined following the movement of water molecules' COM. To test the decoupling approximation, we have computed the ISF from both eq. 1 and eq. 3 and evaluated the difference:

$$\Delta F_s(Q,t) = F_s(Q,t) - F_s^{ROT}(Q,t)F_s^{COM}(Q,t) \tag{4}$$

Figure 5 reports the behavior of the ISF computed via eq. 1 and eq. 3 for a generic high Q-vector in the plastic crystal phase at 8 GPa and 450 K. The blue line represents the behavior of the ISF computed via eq. 1, while the orange line represents the behavior of the ISF as computed via eq. 3. The difference between the two approaches, $\Delta F_s(Q,t)$ (eq. 4) is reported in the inset. It is possible to appreciate how, for times $\lesssim$ 0.3 ps, the two terms are almost identical, while small deviations occur for longer times. In figure 6 we report the $\Delta F_s(Q,t)$ computed over a wide range of Q-vectors and at three pressures, namely P=6 GPa (upper panel), P=7 GPa (middle panel) and P=8 GPa (lower panel). The vertical lines represent the limit of 0.3 ps. We see that, depending on the value of the **Q**-vector, the difference $\Delta F_s(Q,t)$ is between $\sim$ 4% and $\sim$ 6%. We conclude that the decoupling approximation is largely satisfied in the range of stability of plastic ice VII.

2.2 Fitting Simulations

To fit the rotational intermediate scattering functions $F_s^{ROT}(Q,t)$ we constrain the fitting function to the same model adopted to fit the experimental QENS data, i.e. the

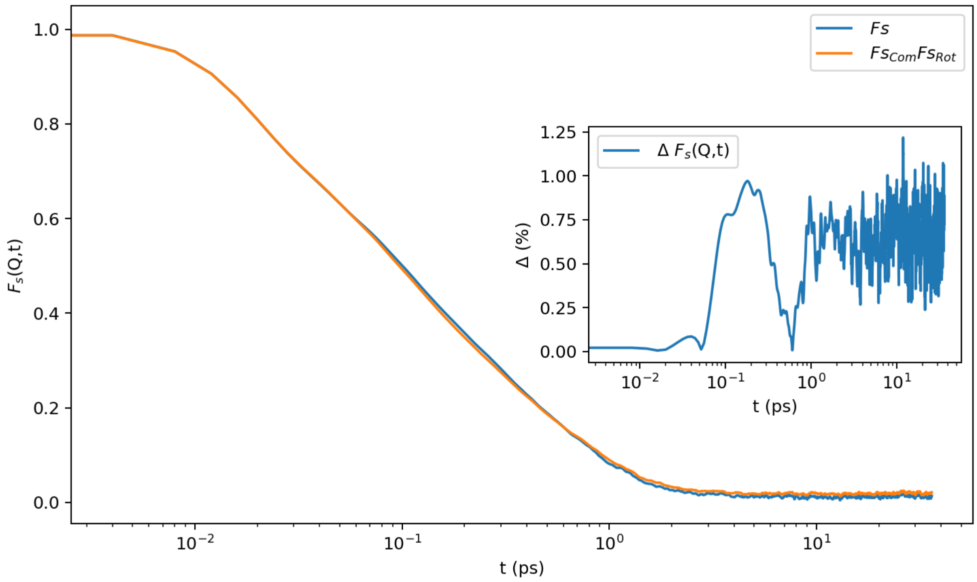

Fig. 5: Example of an ISF accounting both vibrational and rotational contributions (blue line) and the ISF as evaluated via eq. 3 (orange line). The inset reports the difference $\Delta F_s(Q,t)$ between the two curves. The ISFs in this example refer to the thermodynamic condition of 8 GPa and 450 K, calculated for the wave vector corresponding to 2.8 Å^{-1}.

Fourier transform of the cubic tumbling reorientational model, described in the *Methods* section of the main text. This describes rotations that involve the reorientation of hydrogens to other equilibrium positions, to which we add a quadratic contribution to account for the librational motions of the hydrogens:

$$F_s(t,Q) = be^{-(t/\tau_{lib})^2} + (1-b)\left[A_1(aQ)e^{-\frac{2t}{\tau_{90}}} + A_2(aQ)e^{-\frac{4t}{3\tau_{90}}} + A_3(aQ)e^{-\frac{2t}{3\tau_{90}}} + A_0(Q)\right] \tag{5}$$

Figure 7 shows a good agreement in the description of the second decay, related to the rearrangement of the positions of the hydrogens, returning a constant time in Q and close to the value determined by the experimental spectra. Respectively 0.42 ± 0.04 ps for the time obtained in the simulations and 0.56 ± 0.02 ps for the experimental value. Figure 8 presents a comparison of the values assumed by the fit parameters obtained according to eq. 5 with respect to the expected trend for the form factors as defined in the literature [1] and given in eqs. 9, 10 and 11 of the main text. It is assumed that the distance d is equal to 1.13 Å

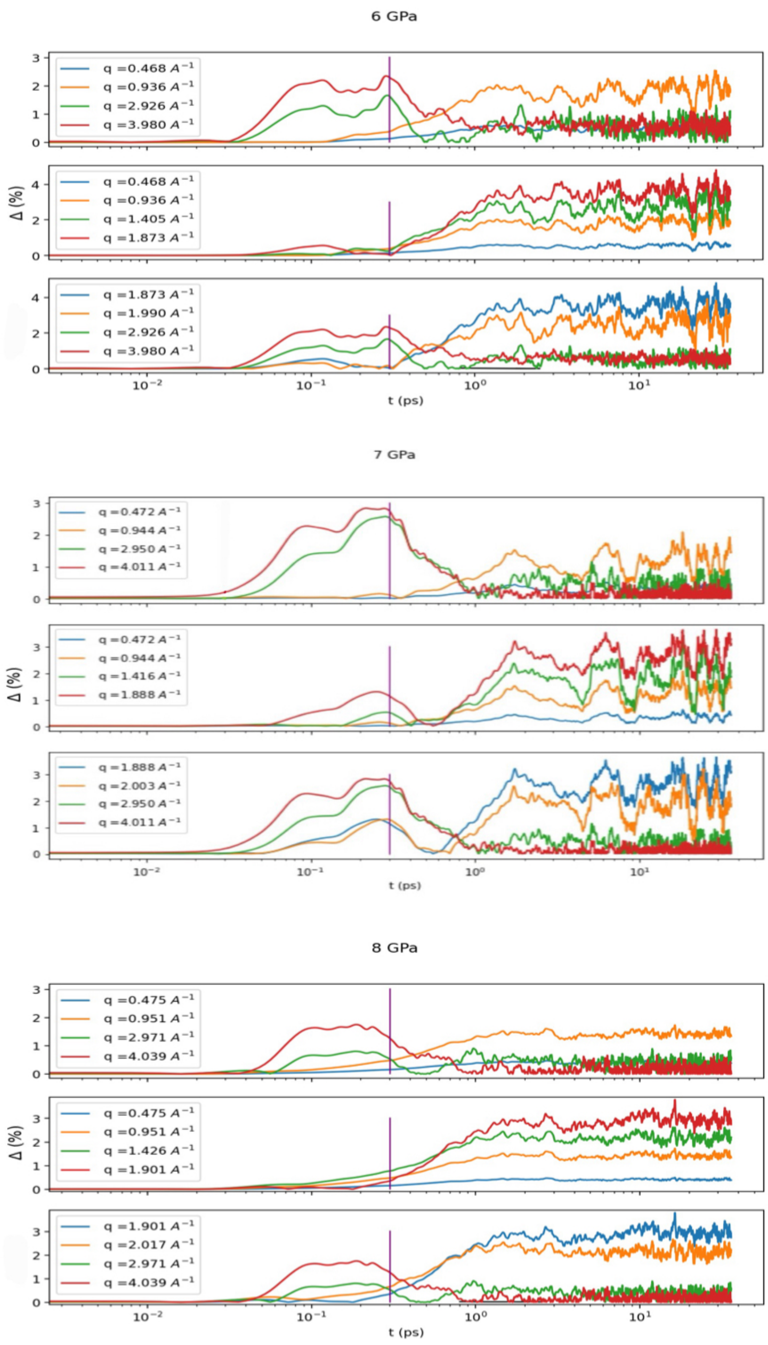

Fig. 6: Difference $\Delta F_s(Q,t)$ computed over a range of Q-vectors and for three pressures: P=6 GPa (upper panel), P=7 GPa (middle panel) and P=8 GPa (lower panel). The vertical lines mark the time 0.3 ps. Below this threshold, $\Delta F_s(Q,t)$ increases as the wave vector increases, while above it increases until it reaches a maximum around 1.9 Å^{-1} and then tends to decrease as the wave vector rises.

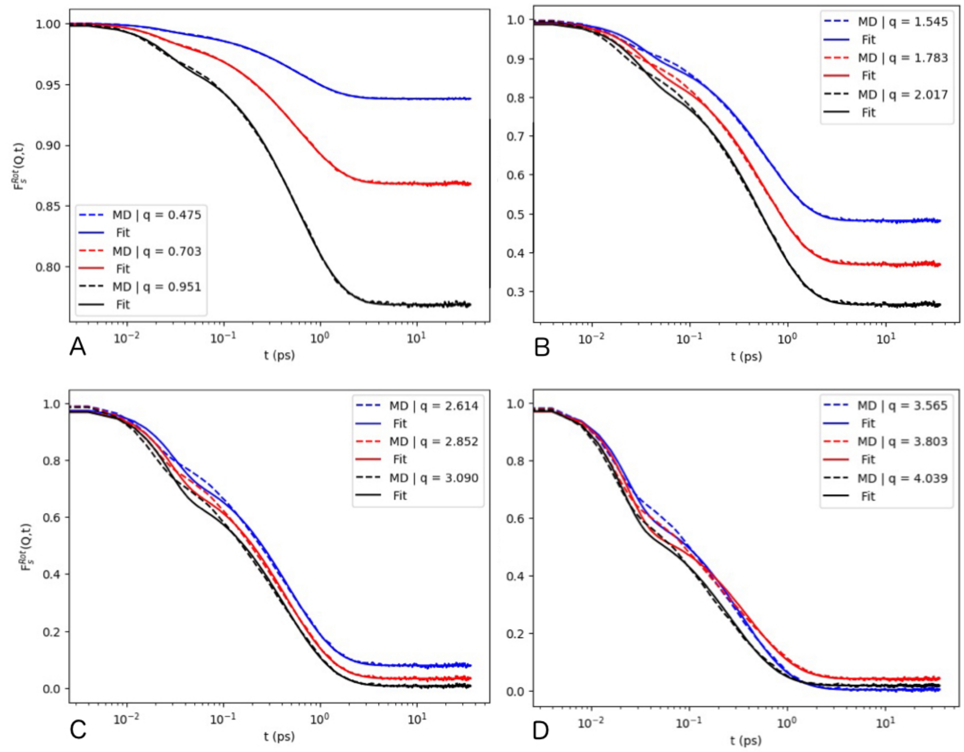

Fig. 7: The figure presents a comparison between ISF spectra derived from eq. 1 (MD) and the fitting function outlined in eq. 5 (Fit).

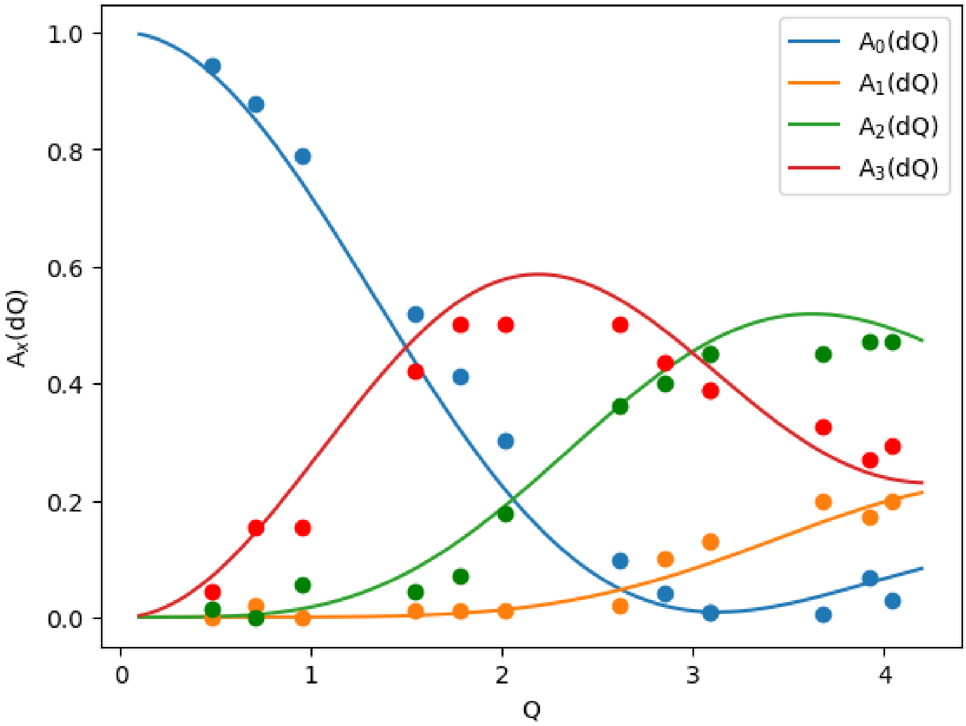

Fig. 8: The figure illustrates the comparison between the values assumed by the fit parameters, as expressed in eq. 5, as a function of the wave vector. The solid dots specify the values assumed for each Q vector plotted in figure 7, while the continuous lines demonstrate the expected trend, assuming that the distance d is equal to 1.13 Å.

2.3 Comparison of the simulated and experimental $S(Q, \omega)$

Figure 9 presents a comparison of the dynamic structure factor obtained from both the experimental data and the simulations. The spectra are representative of the thermodynamic conditions at 518 K and 5.5 GPa for the experimental data and 450 K and 7 GPa for the simulations, respectively. Simulation data are normalized, the normalization constant is chosen in order to obtain a good agreement at high Q, where we know that both simulations and experiments are more reliable. The comparison demonstrates a generally good agreement between the spectra, particularly at higher Q-values. At high Q, the dynamical structure factor is more representative of short-range interactions, which are effectively captured by the simulations. In contrast, at lower Q values, the $S(Q, \omega)$ reflects long-range interactions that may be influenced by sample imperfections in the experimental data, which are not accounted for in the simulations. Additionally, the simulations may be subject to finite-size effects that can impact the accuracy of the results [2].

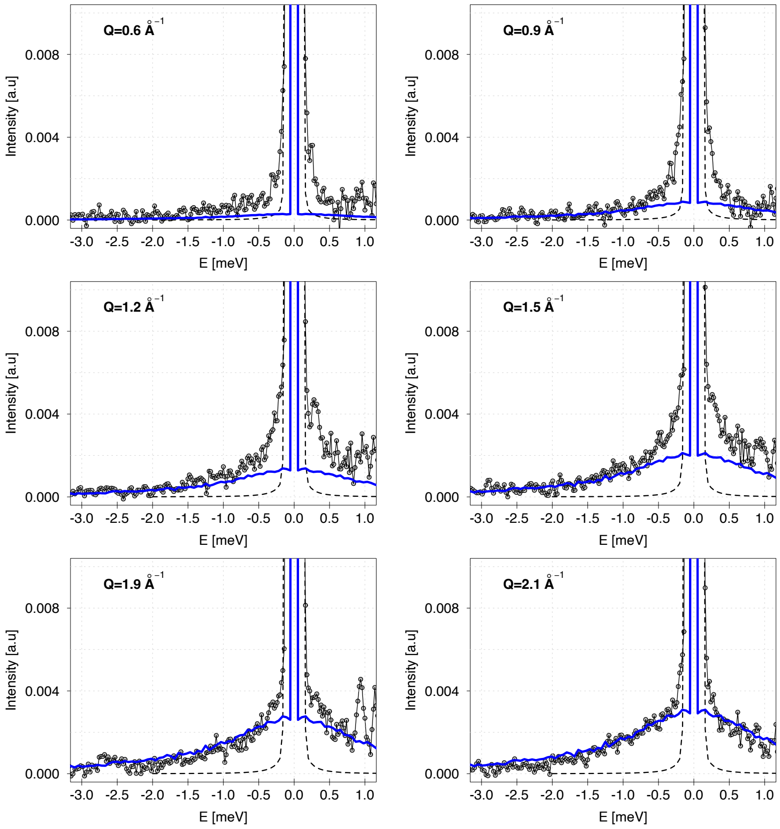

Fig. 9: Comparison of the experimental $S(Q, \omega)$ at 518 K and 5.5 GPa (gray dots and black lines) with simulated spectra (blue lines) at 450 K and 8 GPa. The black dashed line represents intrumental resolution.

References

[1] Remhof, A., Łodziana, Z., Martelli, P., Friedrichs, O., Züttel, A., Skripov, A.V., Embs, J.P., Strässle, T.: Rotational motion of BH_4 units in $M BH_4$ (M=Li,Na,K) from quasielastic neutron scattering and density functional calculations. Physical Review B **81**, 214304 (2010) https://doi.org/10.1103/PhysRevB.81.214304

[2] Salacuse, J.J., Denton, A.R., Egelstaff, P.A.: Finite-size effects in molecular dynamics simulations: Static structure factor and compressibility. I. Theoretical method. Physical Review E **53**, 2382–2389 (1996) https://doi.org/10.1103/physreve.53.2382
